# Post-traumatic stress disorder and depressive symptoms among firefighters: a network analysis

**DOI:** 10.3389/fpubh.2023.1096771

**Published:** 2023-05-04

**Authors:** Peng Cheng, Lirong Wang, Ying Zhou, Wenjing Ma, Guangju Zhao, Li Zhang, Weihui Li

**Affiliations:** ^1^Department of Psychiatry, and National Clinical Research Center for Mental Disorders, The Second Xiangya Hospital of Central South University, Changsha, Hunan, China; ^2^Xiangya School of Medicine, Xiangya Hospital, Central South University, Changsha, China

**Keywords:** post-traumatic stress disorder, depression, network analysis, firefighter, mental health

## Abstract

**Background:**

Firefighters, as first responders with a high risk of occupational exposure to traumatic events and heavy working stress, have a high prevalence of PTSD symptoms and depressive symptoms. But no previous studies analyzed the relationships and hierarchies of PTSD and depressive symptoms among firefighters. Network analysis is a novel and effective method for investigating the complex interactions of mental disorders at the symptom level and providing a new understanding of psychopathology. The current study was designed to characterize the PTSD and depressive symptoms network structure in the Chinese firefighters.

**Method:**

The Primary Care PTSD Screen for DSM-5 (PC-PTSD-5) and the Self-Rating Depression Scale (SDS) were applied to assess PTSD and depressive symptoms, respectively. The network structure of PTSD and depressive symptoms was characterized using “expected influence (EI)” and “bridge EI” as centrality indices. The Walktrap algorithm was conducted to identify communities in the PTSD and depressive symptoms network. Finally, Network accuracy and stability were examined using the Bootstrapped test and the case-dropping procedure.

**Results:**

A total of 1,768 firefighters were enrolled in our research. Network analysis revealed that the relationship between PTSD symptoms, “Flashback” and “Avoidance,” was the strongest. “Life emptiness” was the most central symptom with the highest EI in the PTSD and depression network model. Followed by “Fatigue” and “Interest loss.” Bridge symptoms connecting PTSD and depressive symptoms in our study were “Numb,” “High alertness,” “Sad mood,” and “Compunction and blame,” successively. The data-driven community detection suggested the differences in PTSD symptoms in the clustering process. The reliability of the network was approved by both stability and accuracy tests.

**Conclusion:**

To the best of our knowledge, the current study first demonstrated the network structure of PTSD and depressive symptoms among Chinese firefighters, identifying the central and bridge symptoms. Targeting interventions to the symptoms mentioned above may effectively treat firefighters suffering from PTSD and depressive symptoms.

## 1. Introduction

Post-traumatic stress disorder (PTSD) is a chronic severe mental disorder after traumatic exposure, which is composed of four core symptoms, intrusion symptoms associated with the traumatic events, avoidance of traumatic stimuli, negative alterations in cognitions and mood, and marked alterations in arousal and reactivity (1). Given that over 70% of people may experience a traumatic event in their lifetime (2), previous studies indicate that people with PTSD symptoms are more likely to have other psychiatric symptom comorbidities (3), especially depressive symptoms (4). The high rate of symptom co-occurrence of PTSD and depression is demonstrated in populations with different categories (natural disasters, combat violence, the break of an intimate relationship, etc.) of traumatic exposures (5–8). Either PTSD symptoms or depressive symptoms are associated with higher final mortality and morbidity of physical symptoms [musculoskeletal pain, cardio-respiratory (CR) symptoms, gastrointestinal (GI) symptoms, etc.] (9, 10). Moreover, emerging research suggests that the co-occurrence of PTSD and MDD possibly has more severe adverse effects on mental health and life quality than either of them alone (11).

Firefighters, first responders with a high risk of occupational exposure to traumatic events and heavy working stress (12), are the population with a high prevalence of PTSD symptoms and depressive symptoms. A systematic review suggests that the mean prevalence of PTSD among firefighters is 12.3%, and the mean prevalence of depression is 18.7%. Both of them are higher than those of the community population (13). Since stressful and frightening working situations are almost inevitable for firefighters, the potential risk of a series of negative results caused by PTSD and depression is extremely serious, including alcohol abuse, occupation burnout, and even suicidal ideation or attempt (14–16). Besides, from the clinical perspective, there is a substantial amount of symptoms overlap between PTSD and depression, like sleep disturbance, inattention, avoidance, and withdrawal, etc. Diagnostic confusion brought by the similarity of symptoms is common, especially when clinicians lack information of traumatic event history (17). The concealment of psycho-trauma related information is not unusual in the Asian population, as high levels of stigma are general in the Asian culture and mental disorders are regarded as a sign of weakness (18). Therefore, we consider that it is meaningful to figure out the potential relationships and significance hierarchy among symptoms of PTSD and depression, which can provide references for accurate psychological intervention for firefighters.

In recent years, network analysis has become a widely applied method for exploring the associations among individual psychiatric symptoms and ascertaining their relative significance in psychopathology (19). Network analysis posits that psychiatric disorders/syndromes can be modeled as phenomena arising as a complex network of interacting and mutually reinforcing symptoms (20). Thus, specific symptoms act as positive roles in activating and maintaining mental disorders instead of passive indicators of mental disorders (21). Compared with the traditional statistical analysis method mainly investigate the unidirectional association (e.g., structural equation modeling, regression analysis, etc.) (22, 23), which demands a hypothesis of the potential relationships among symptoms, network analysis can identify the dynamic and reciprocal associations between various symptoms (24, 25).

Network analysis can provide the corresponding centrality and predictability index for each symptom node to test its significance and controllability in the whole network (26, 27). The centrality index can be used to ascertain central symptoms which contribute to the majority of the network stability, for further understanding mechanisms about the onset and maintenance of a disorder or syndrome and potential targets for clinical interventions (24). Moreover, when individuals suffer from different psychiatric disorders, some specific symptoms of one disorder may increase the risk of the other disorder, which is considered as bridge symptoms in the network. The bridge symptoms of the network play essential roles in maintaining and developing comorbidities, suggesting references for clinical prevention and treatments (28).

Previous studies of the network of the comorbidity of PTSD and depression were mainly conducted in veterans (29, 30), though heterogeneity exists between different studies, indicating higher centralities of symptoms “Flashback,” “Getting upset by trauma reminders,” “concentration difficulties,” and “anhedonia.” Another network analysis enrolling male participants with domestic violence indicates that symptoms “Feelings of worthlessness” and “Avoiding internal reminders of the traumatic experiences” are the most central symptoms (31). To the best of our knowledge, similar studies involving the comorbidity of PTSD and depression are rarely conducted in firefighter samples. Concerned about the difference in exposure to traumatic events and the potential effect of stigma rooted in Asian culture on mental symptoms, we speculated that features of the network of comorbidity of PTSD and depressive symptoms in Chinese firefighters might differ from those of other kind populations in the background of western culture.

As far as we know, the current study is the first study applying the network analysis method to investigate the network structure of comorbidities of PTSD symptoms and depressive symptoms among Chinese firefighters, which inspired us to conduct this study to fill the gap. The purpose of our study was to examine the associations between PTSD and depressive symptoms among Chinese firefighters, then to figure out the center symptoms and bridge symptoms of this PTSD-depressive symptoms network. Our results can provide theoretical references for establishing precise psychological interventions and normalized mental health care for firefighters.

## 2. Materials and methods

### 2.1. Participants and study design

Our study was a cross-sectional survey conducted in March 2021 in Changsha, Hunan Province, China. The Web-based questionnaire was applied in our research for higher data collection efficiency and lower risk of transmission of COVID-19. The questionnaire was set to be qualified for uploading when all items were filled correctly and checked by software, which could improve the reliability of raw data. After being approved by the administration of Hunan Fire Brigade, electronic questionnaires set by us were distributed by the management department of Hunan Fire Brigade. Inclusion criteria were (1) age above 18 years old, (2) certificated firefighters in fire stations, and (3) understanding the purpose and content of this research. Exclusion criteria were (1) not being at work due to any reasons, (2) not able to finish the whole questionnaire due to health reasons, and (3) without any experience of formal emergency mission. This study was approved by the Ethic Committee of the Second Xiangya Hospital of Central South University. All the participants have provided electronic written informed consent.

### 2.2. Measures

The primary care PTSD screen for DSM-5 (PC-PTSD-5) was used for assessing PTSD symptoms. PC-PTSD-5 is a five item self-report screening scale, which is set based on the diagnostic criteria of PTSD in DSM-5 (32). Items are scored dichotomously as either zero or one (0 = No; 1 = Yes). Higher scores mean more severe PTSD symptoms. The reliability of the Chinese version of PC-PTSD-5 in Chinese was validated and the cutoff score of potential PTSD was set as two points (33).

Depressive symptoms were assessed by the Self-Rating Depression Scale (SDS), which was compiled by Zung et al. (34). SDS is a classical and widely applied four-point Likert scale for depression in China (35), scored from one (a little of the time) to four (most of the time). Higher scores mean more severe depressive symptoms. According to the SDS Chinese manual (36), individuals with a total score of 40 or more were considered to have potential depression.

### 2.3. Statistical analysis

All the data were analyzed by the R program (37). The process of network analysis was divided into five domains, including network estimation and visualization, Exploratory community analysis, centrality and predictability analysis, network accuracy and stability, and network comparison.

#### 2.3.1. Network estimation and visualization

Partial correlation analysis was applied to analyze the association between each pairwise nodes for controlling the confounding effects of other nodes in the network. The least absolute shrinkage and selection operator (LASSO) algorithm was used in the regularization process to shrink all edges in the network and set small correlations to zero, which enabled as few nodes and edges as possible in the network (38). Meanwhile, the extended Bayesian Information Criteria (EBIC) were also adopted to obtain a sparse and interpretable network model (39). The turning parameter was set for 0.5, which was widely applied for controlling spurious correlations in the network estimation (40).

Fruchterman-Reingold algorithm (41) was used to visualize the network. Nodes with stronger and more frequent associations with other nodes were placed closer together and more centralized in the network.

According to the parlance of network analysis, each item represented a symptom node, and each edge from one node to another was indicated as the association between two nodes. Thicker and more saturated edges meant stronger relationships. The color of edges represented the correlation, generally green for positive and red for negative (42). Concerned that the primary purpose of the current study was to investigate the inducing relationships between PTSD and depressive symptoms, and also for the simplicity and better understanding of the network structure, only positive relationships were depicted in the visualization of network. The possible negative associations among nodes were retained in the correlation matrix for the integrity of data. The R packages *bootnet* (Version 1.5) (43) and *qgraph* (Version 1.9.2) (42) were used to estimate and visualize the network.

#### 2.3.2. Exploratory community analysis

Concerned that actual dimensions of symptoms in the PTSD and depression network might differed from initial hypothetical dimensions, we conducted the data-driven method, community detection, to identify the final dimensions of symptoms in the network. Meanwhile, the centralities (both original and bridge) of the final community detection grouping were calculated to be compared with the centralities of the original hypothetical grouping.

The Walktrap algorithm, based on the principle that adjacent nodes tend to belong to the same community (44), was applied to identify communities in the network model (45). This algorithm has been proven that performs well on psychological networks (46, 47). The function *cluster_walktrap*, available in the R package *igraph* was used to detect communities in the current study.

#### 2.3.3. Centrality and predictability analysis

The centrality index, expected influence (EI), was calculated by the R package *qgraph* (1.9.2) (42) to quantify the importance of each symptom node in the network model. Symptom node EI refers to the sum of the value of all edges connecting to a given node, including both positive and negative values (48). Since there were not only positive associations but also negative associations in the network of our study, EI is more appropriate than other centrality indexes. Moreover, the bridge EI was analyzed to evaluate the significance of a node in connecting external symptom dimensions, by using the function *bridge* of the R package *networktools* (version 1.4.0) (49).

Predictability, an index suggesting the extent to which a node was predicted by its neighboring nodes (39), was computed by the function *predict* of the R package *mgm* (version 1.2–12) (50). Predictability represents the controllability of a node (27). In the picture of network model, the ring area around each node illustrated the value of predictability.

#### 2.3.4. Network accuracy and stability

A total of three steps were conducted by the R package *bootnet* (version 1.5) (43) to assess the accuracy and stability of the network model in our research. Firstly, the accuracy of edge weights was estimated by constructing a 95% confidence interval (CI) with non-parametric bootstrapping method (51), which was visualized with a line diagram. A narrower bootstrapped CI of edge weights suggested a more precise estimation of the edges (52).

Secondly, the stability of symptom node EI was tested by calculating the correlation stability coefficient (CS-C), using a case-dropping bootstrap procedure (53). In this procedure, increasing percentage of cases was dropped from the dataset, and the EI indexes were re-estimated. If the EI of symptoms nodes did not change significantly after excluding a subset of the sample, the network structure could be considered stable. CS-C means the maximum proportion of samples that could be dropped while the EI correlation between the networks of original sample and case-dropping subsets was at least 0.7 with a 95% probability (43). In general, the value of CS-C needs to be above 0.25 and is preferably above 0.5 (43).

Thirdly, the significant differences between edge weights and node EI centralities were computed using the bootstrapped difference tests based on 95% CIs, which suggested that there were statistical differences between two edges weights or two EI centralities if zero was not included in the CIs (43).

#### 2.3.5. Network comparison

Only-child status was collected as demographic information in the current study, according to which network comparison analysis was conducted to investigate the difference in network structure between only-child firefighters and non-only-child firefighters. China is a country with a huge population, family planning policy was conducted by the government so far, to control the rapid increase of population. Concerned that previous studies proved the effect of only-child status on mental health (54–56), we speculated that only-child and non-only-child firefighters might have a different network structure from each other.

The function *network comparison test (NCT)* of the R package *NetworkComparisonTest* (57) was applied to compare the network structures. The principle of NCT was a permutation-based test, which randomly regroups participants from each sub-network repeatedly (1,000 times) and then calculates the differences between networks. A total of three invariance measures were examined of sub-networks of genders, including global strength, network structure, and edge strength (57). The global strength tested the difference in overall network connectivity between sub-networks. The network structure examined the overall difference between all the possible edges between two sub-networks. Only when a statistically significant difference was found in either global strength or network structure comparisons, edge strength was further calculated to investigate the possible difference in each edge between the two networks. Moreover, the original centralities of nodes between sub-networks were also tested for the completeness of analysis.

## 3. Results

### 3.1. Descriptive statistics

A total of 1,781 firefighters were included in our study. For the demographic information, all of the participants were male, 624 (35.0%) of which were only-child and 1,157 were non-only-child. The average age of sample was 26.60 [*standard deviation* (SD) = 4.82]. The average total scores were 0.34 (*SD* = 0.83) for PC-PTSD-5 and 36.21 (*SD* = 9.34) for SDS. According to the cutoff scores applied in the current research, the proportions of firefighters with potential PTSD and potential depression were 8.7 and 36.3%, respectively. The mean score and abbreviation for each symptom node are shown in Table 1.

**Table 1:** Descriptive statistics of PTSD and depressive symptom nodes.

Abbreviation	Symptom node	Mean (SD)
PCPTSD1	Flashback	0.07 (0.26)
PCPTSD2	Avoidance	0.06 (0.24)
PCPTSD3	High alertness	0.09 (0.29)
PCPTSD4	Numb feeling	0.07 (0.26)
PCPTSD5	Compunction and blame	0.04 (0.20)
Total score of PC-PTSD-5		0.34 (0.83)
SDS1	Sad mood	1.25 (0.49)
SDS2	Mood circadian rhythm	2.74 (1.08)
SDS3	Crying	1.08 (0.28)
SDS4	Sleep	1.51 (0.69)
SDS5	Appetite	2.49 (1.17)
SDS6	Sexuality	2.54 (1.17)
SDS7	Weight loss	1.26 (0.51)
SDS8	constipation	1.16 (0.44)
SDS9	Tachycardia	1.14 (0.38)
SDS10	Fatigue	1.30 (0.55)
SDS11	Confusion	2.23 (1.23)
SDS12	Ability decline	2.53 (1.19)
SDS13	Feeling of upset	1.21 (0.47)
SDS14	Hopelessness	2.02 (1.16)
SDS15	Irritability	1.25 (0.50)
SDS16	Irresolution	2.83 (1.01)
SDS17	Unworthiness	2.25 (1.13)
SDS18	Life emptiness	2.15 (1.13)
SDS19	Guilty	1.06 (0.29)
SDS20	Interest loss	2.21 (1.17)
Total score of SDS		36.21 (9.34)

PCPTSD, the primary care PTSD screen for DSM-5; SDS: the Self-Rating Depression Scale; and SD: standard deviation.

### 3.2. Network structure

**Figure 1:**
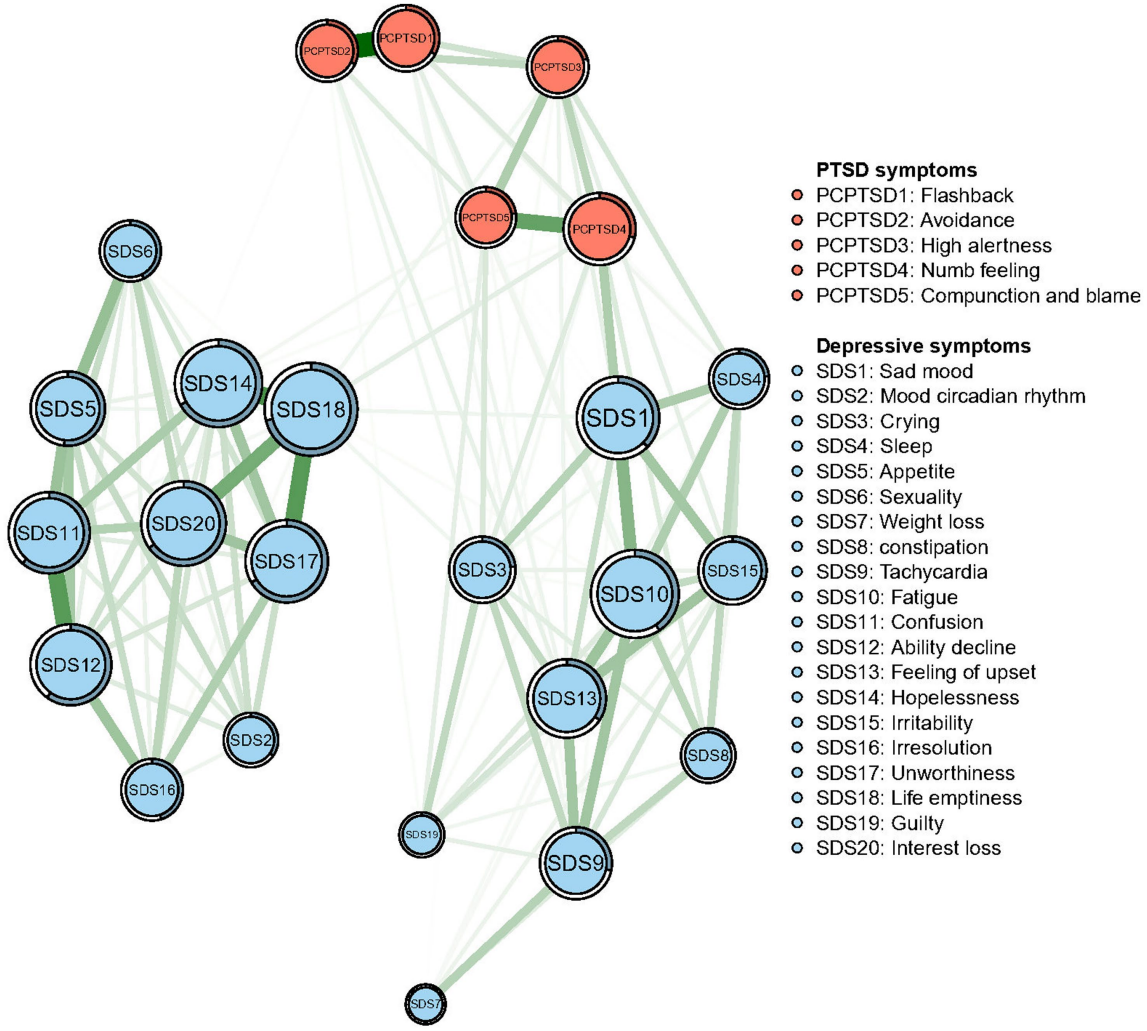
Network structure of post-traumatic stress disorder (PTSD) and depressive symptoms in Chinese firefighters. Symptom nodes with stronger associations are placed closer to each other. The dark green lines represent positive correlations. The red lines represent negative correlations. The line thickness represents the strength of the connection between symptom nodes. Ring-shaped pie charts illustrate predictability (e.g., a fully filled dark ring means that 100% of the symptom’s variance can be explained by its intercorrelations with the other symptom nodes in the network).

The network of PTSD and depressive symptoms is depicted in Figure 1. The detailed Correlation matrix is represented in Supplementary Table 1.

A total of 300 edges [25 × (25–1)/2] were initially estimated, of which 140 edges were non-zero weights and entered further analysis. For the connection between symptoms, “Flashback” (PCPTSD1) and “Avoidance” (PCPTSD2) were the strongest edges (weight = 0.46), followed by the edges between “Hopeless” (SDS14) and “Life emptiness” (SDS18) (weight = 0.33), between “Unworthiness” (SDS17) and “Life emptiness” (SDS18) (weight = 0.30), and between “Confusion” (SDS11) and “Ability decline” (SDS12) (weight = 0.30).

In terms of the predictability, the node “Life emptiness” had the highest predictability value (71.1%), while the mean level of predictability of nodes was 38.3 ± 18.2%. These results meant this symptom node was the most central symptom in the PTSD-depression interactive network but also the node highest-predicted by surrounding nodes.

### 3.3. Community and centrality

**Figure 2:**
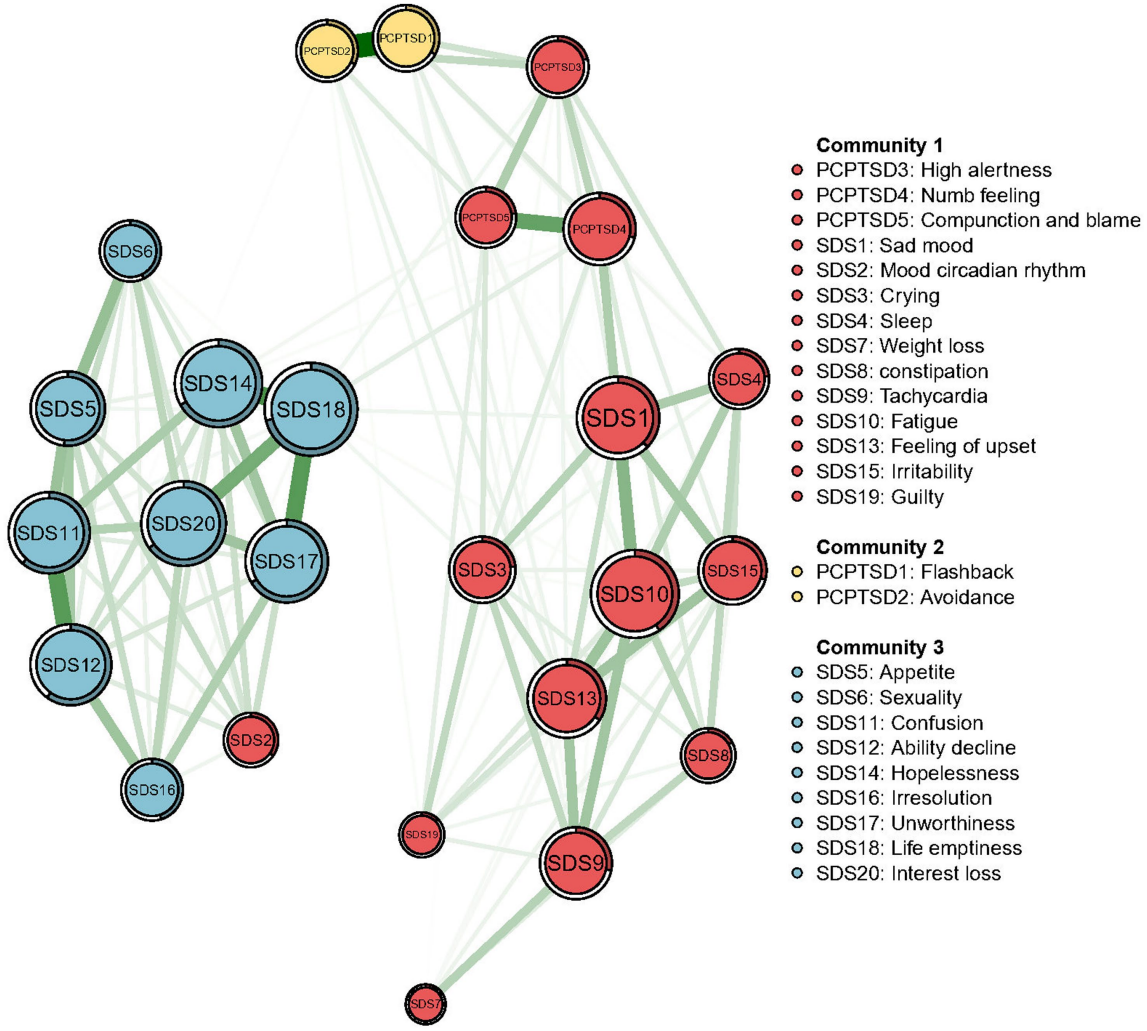
Communities detected by the Walktrap algorithm in the network of PTSD and depressive symptoms.

The Walktrap algorithm detected three communities in the comorbidity of PTSD and depressive symptoms (Figure 2). “Flashback” (PCPTSD1) and “Avoidance” (PCPTSD2) remained in the community of PTSD (Community 2), while “High alertness” (PCPTSD3), “Numb feeling” (PCPTSD4), and “Compunction and blame” (PCPTSD5) were included in the Community 1 with depressive symptoms. The Community 3 was entirely generated by the rest depressive symptoms.

**Figure 3:**
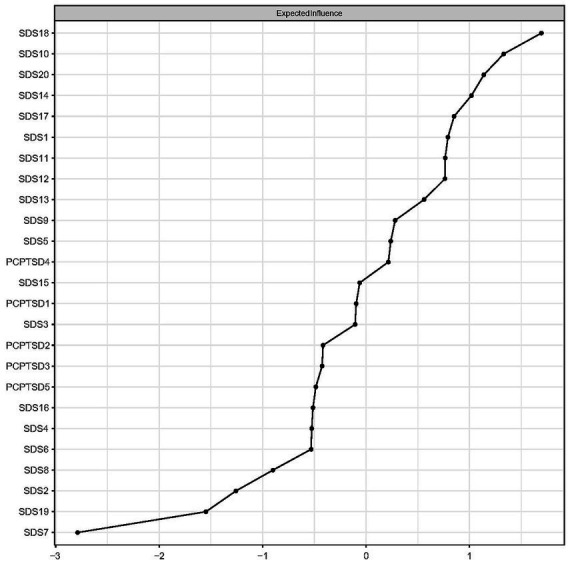
Expected influences of PTSD and depressive symptom nodes in the network (shown as standardized values *z* scores).

All centralities (original EIs and bridge EIs) based on the theoretical model and data-driven detected model were both calculated for comparison. Details are shown in Table 2. As the original EI, measuring the significance of a certain node in the whole network, was not affected by the dimension distribution. Thus, original EIs based on the theoretical model were as same as the data-driven detected model, which are shown in Figure 3. The depressive symptom, “Life emptiness” (SAS18), had the highest EI value, indicating that this symptom was the most central symptom in the network model, which was followed by the symptom “Fatigue” (SDS 10), “Interest loss” (SDS 20), and “Hopeless” (SDS 14).

**Table 2:** Original centralities and bridge centralities of PTSD and depressive nodes in the network.

Symptom node	Abbr.	Predictability	Original EI	Bridge EI of theoretical model^a^	Bridge EI of community-detected model^b^		
					Community 1 vs. others	Community 2 vs. others	Community 3 vs. others
Flashback	PCPTSD1	0.330	−0.095	0.127	0.315	0.315	0
Avoidance	PCPTSD2	0.320	−0.415	0.08	0.239	0.244	0.005
High alertness	PCPTSD3	0.207	−0.425	0.235	0.221	0.194	0.027
Numb feeling	PCPTSD4	0.288	0.216	0.384	0.108	0.06	0.048
Compunction and blame	PCPTSD5	0.228	−0.485	0.165	0.12	0.099	0.022
Sad mood	SDS1	0.373	0.792	0.191	0.007	0	0.007
Mood circadian rhythm	SDS2	0.348	−1.258	0	0.55	0	0.55
Crying	SDS3	0.234	−0.106	0.161	0.053	0.027	0.026
Sleep	SDS4	0.235	−0.525	0.11	0.025	0.025	0
Appetite	SDS5	0.516	0.239	0	0.079	0	0.079
Sexuality	SDS6	0.427	−0.531	0	0.067	0	0.067
Weight loss	SDS7	0.097	−2.789	0.003	−0.06	0	−0.06
constipation	SDS8	0.170	−0.9	0.09	0.024	0.024	0
Tachycardia	SDS9	0.282	0.281	0.039	0.022	0.022	0
Fatigue	SDS10	0.406	1.331	0.076	0.032	0.025	0.007
Confusion	SDS11	0.614	0.765	0	0.034	0	0.034
Ability decline	SDS12	0.600	0.763	0.005	0.043	0.005	0.047
Feeling of upset	SDS13	0.342	0.561	0.099	0.051	0.069	−0.018
Hopelessness	SDS14	0.668	1.019	0.031	0.09	0	0.09
Irritability	SDS15	0.293	−0.062	0.048	−0.007	0.002	−0.009
Irresolution	SDS16	0.452	−0.513	−0.009	0.018	0	0.018
Unworthiness	SDS17	0.664	0.851	0	0.002	0	0.002
Life emptiness	SDS18	0.711	1.695	0.075	0.233	0	0.233
Guilty	SDS19	0.124	−1.548	0.073	0.008	0.008	0
Interest loss	SDS20	0.652	1.139	0	0.035	0	0.035

PCPTSD, the primary care PTSD screen for DSM-5; SDS, the self-rating depression scale; Abbr., abbreviation; EI, expected influence.

a Symptom nodes in the network were pre-divided into two community (PTSD and depressive) based on the specific content of each item in the measurement. Details in Figure 1.

b Symptom nodes in the network were divided into three communities (1,2, and 3) based on the results of data-driven analysis. Bridge EIs were calculated for each community and the other nodes in the network, respectively. Details in Figure 2.

**Figure 4:**
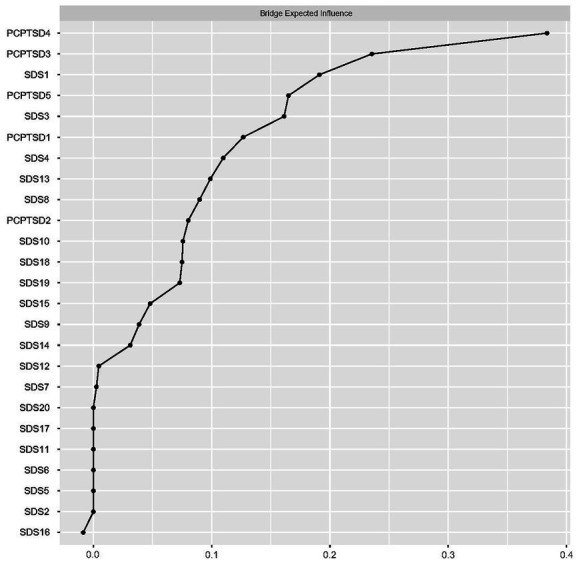
Bridge expected influences of PTSD and depressive symptom nodes in the theoretical model (shown as standardized values *z* scores).

Regarding the bridge EI, in the theoretical model, “Numb feeling” (PC-PTSD-4) had the highest bridge EI, followed by “High alertness” (PC-PTSD-3), “Sad mood” (SDS1), and “Compunction and blame” (PC-PTSD-5). Details are shown in Figure 4.

**Figure 5:**
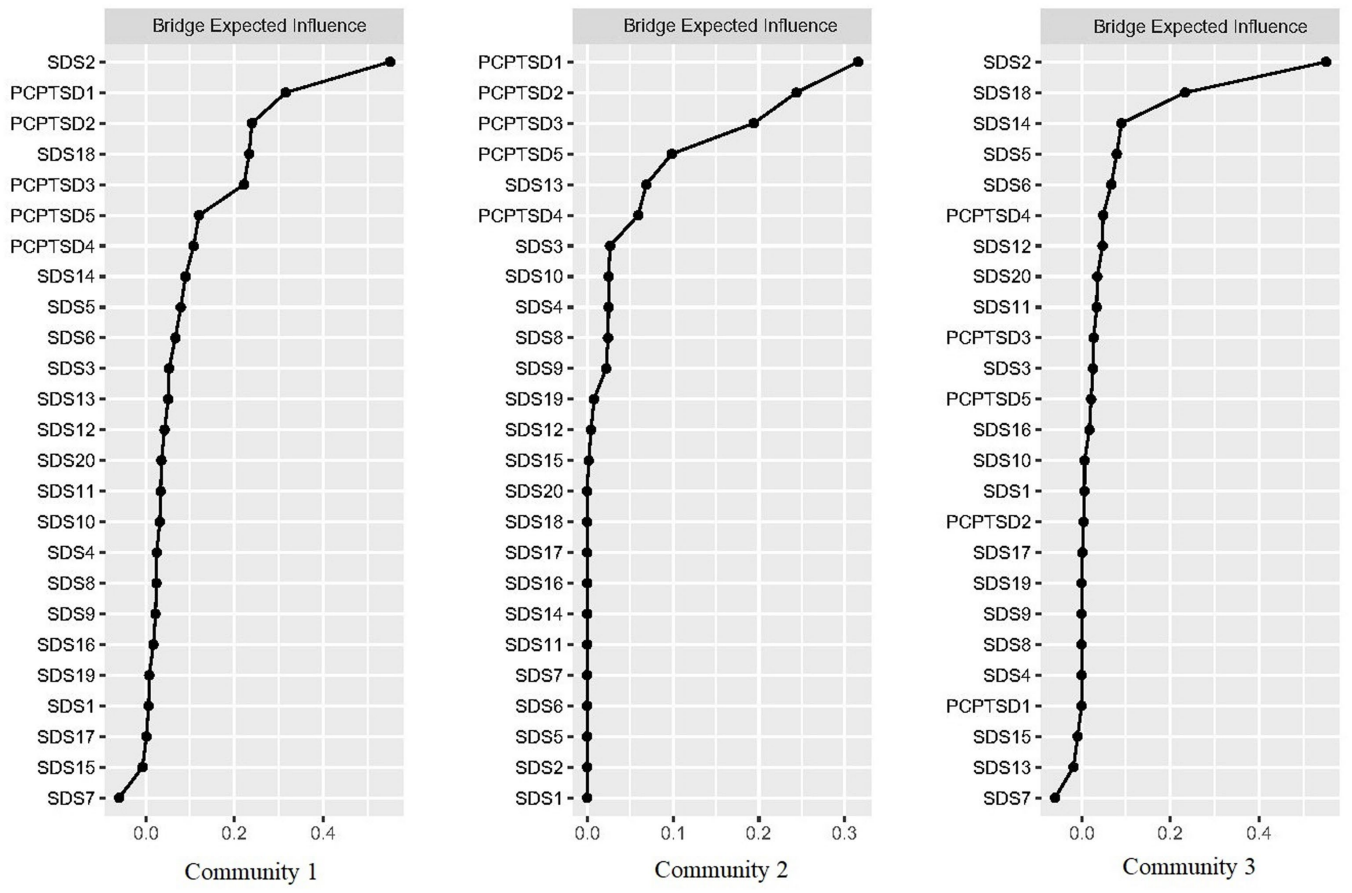
Bridge expected influences of PTSD and depressive symptom nodes in the data-driven model (shown as standardized values *z* scores).

In the data-driven model (detected by the Walktrap algorithm), the bridge EIs of symptom nodes were calculated for each community (Figure 5). In community 1, “Mood circadian rhythm” (SDS2) was the node with the highest bridge EI, being significant in connecting external nodes. In community 2, “Flashback” (PCPTSD1) was the node with the highest bridge EI. In community 3, “Life emptiness” (SAS18) had the highest bridge EI.

### 3.4. Network accuracy and stability

**Figure 6:**
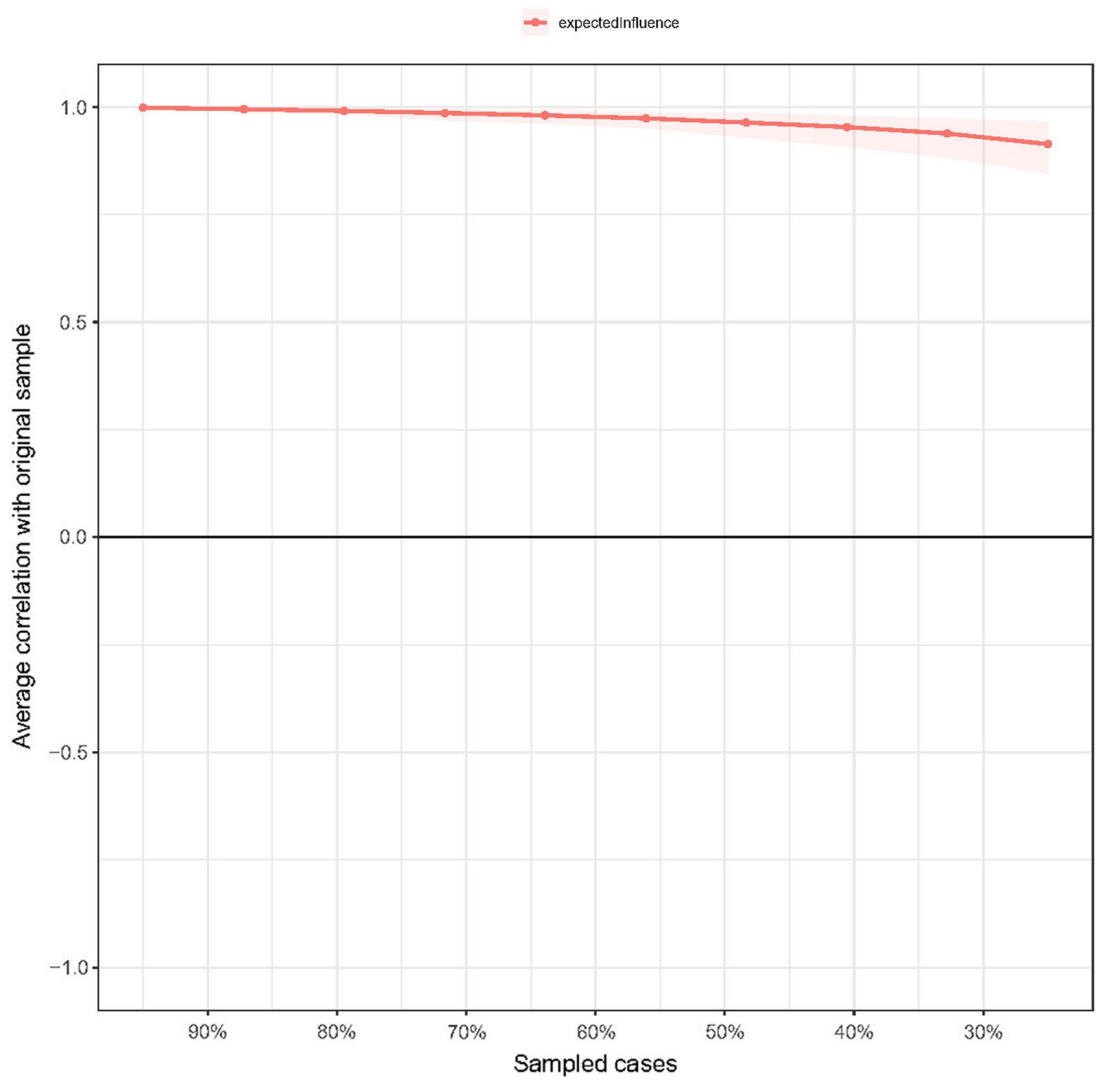
Stability of expected influence using case-dropping bootstrap. The *x*-axis represents the percentage of cases in the original sample used at each step. The *y*-axis represents the average of correlations between the expected influence in the original network and the expected influence in the networks that were re-estimated after dropping increasing percentages of cases.

**Figure 7:**
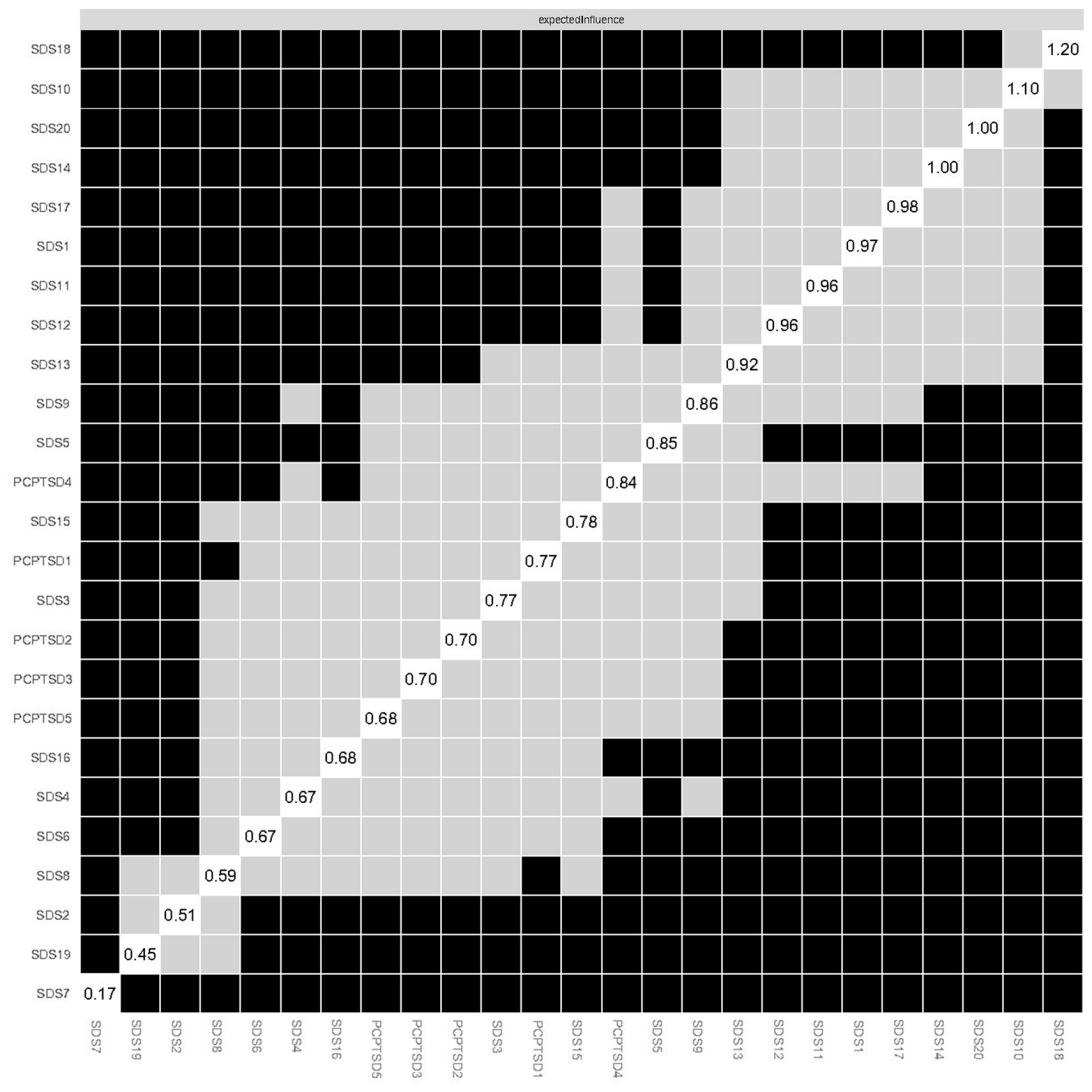
Estimation of node expected influence difference by bootstrapped difference test. Gray boxes indicate nodes that do not significantly differ from one another. Black boxes represent nodes that differ significantly from one another. Values in box on the diagonal represent the expected influence of each node.

For the accuracy of edge weight, as shown in Supplementary Figure 1, bootstrap 95% CIs were narrow, indicating that the estimations of edges were reliable. As presented in Figure 6, the case-dropping bootstrap procedure indicates that the CS-C of EI was 0.75, reflecting that the network kept being stable after dropping 75% of the sample. In the Bootstrapped difference tests for the node EIs (Figure 7), central symptom nodes differed from most other nodes. And the Bootstrapped difference tests for edge weights (Supplementary Figure 2) also showed that most comparisons between edges were statistically different, especially the strongest edges.

### 3.5. Network comparison

The network structures for both only-child firefighter sub-network and non-only child firefighter sub-network are, respectively, shown in Supplementary Figures 3, 4. The network comparison test (NCT) did not find statistically significant difference in the global strength invariance between only-child firefighters and non-only-child firefighters (only-child: 10.497 vs. non-only-child: 9.688, Test statistic *S* = 0.809, *p* = 0.125). Meanwhile, there was no significant difference found in network structure invariance (Test statistic *M* = 0.175, *p* = 0.311). No significant difference of original centrality was detected in the comparison of only-child sub-network and non-only child sub-network (Test statistic *C* = 0.133–0.468, *p* = 0.20–0.99). Details of original centrality comparison are shown in Supplementary Table 2.

## 4. Discussion

To the best of our knowledge, the current study is the first study focusing on characterizing the network structure of PTSD and depressive symptoms among the firefighters in China. For the associations between symptom nodes, there were no cross-dimension edges in the strong edges. The strongest edge was the connection between “Flashback” and “Avoidance” within the PTSD sub-network.

Compared with a previous study conducted in veterans in which the edge between PTSD and depressive symptoms was the strongest in the whole network (29), our results suggested that the association between “Flashback” and “Avoidance” is the strongest connection, even in the symptom network simultaneously including PTSD and depressive symptoms. We speculated that our results revealed the effect of PTSD symptoms in the conjunct network was more limited in firefighters. Thus, the risk of inducing depressive symptoms caused by traumatic-related symptoms was lower.

According to the node EI and predictability results, “Life emptiness” was the most central symptom with the highest EI and the symptom node with the highest predictability. Feeling of emptiness, which could be described as a sense of deadness or absence of inner feelings, is a complexed and negative emotion, including physical component, aloneness component and component involving personal unfulfillment, and lack of purpose (58, 59). Feeling of emptiness was observed in people with depression (60) and PTSD (61), thus, it should be recognized as a transdiagnostic construct. Additionally, previous studies demonstrated that the comorbidity of PTSD and depression could be explained by similar psychopathology symptom dimensions across these two illnesses. And PTSD and depression might present a same traumatic stress construct with common related factors, especially in chronic cases (4). Based previous results, the current study further demonstrated that “feeling of emptiness” was the most significant factor in activating and stabilizing the PTSD-depression psychopathology network. Concerned the high incidence of PTSD and depression comorbidity (17, 62, 63), it is meaningful to figure out the core symptom node in the PTSD-depression network and manipulate accurate intervention aiming “feeling of emptiness” could simultaneously alleviate both PTSD and depressive symptoms.

As for the bridge symptoms, from the perspective of the association between PTSD symptoms and depressive symptoms (i.e., the theoretical model), the most influential bridge symptom node was the “Numb feeling.” Numb feeling was considered as one of the hallmark symptoms of PTSD, including *markedly diminished interest in significant activities* (*Criterion C-4*), *feelings of detachment or estrangement from others* (*C-5*), *and restricted range of affect* (*C-6*), which were represented in the diagnostic criteria of PTSD of DSM-IV (64). Meanwhile, anhedonia is a symptomatic deficit in positive affect, involving the loss of enjoyment in pleasurable activities or the loss of desire to engage in them. This is similar to the Criterion C-4 criterion of numb feeling. Anhedonia was considered to be related to the core symptom of depression and was so common that approximately one-third of depressed individuals have clinically significant anhedonia symptoms (65). Based on the previous studies and the results of our study, we speculated that our results demonstrated the “Numb feeling” acted as “mediating node” between the PTSD symptom cluster and the depressive symptom cluster in the Chinese firefighters, a population with a high risk of traumatic exposure. Additionally, an emotional dysregulation model of PTSD illustrated by Litz et al. (66) held that the “emotionally numb” of individuals with PTSD was actually a result of hyperresponsivity to negative emotional stimulation, individuals with PTSD required more intense positive stimulation to access pleasure. Thus, our results verified this theoretical model among Chinese firefighters from the network analysis perspective. Concerned depressive symptoms usually followed or co-occurred with the PTSD symptoms, instead of existing alone in the context of the traumatic exposure, psychological intervention targeting at “Numb feeling” might control the comorbidity of PTSD and depression.

Notably, the bridge symptoms in the network were also supported by the results of data-driven model. “High alertness,” “Numb feeling,” and “Compunction and blame,” three PTSD symptom nodes with relatively high bridge EI were divided into a community with some depressive symptom nodes. While “Flashback” and “Avoidance” composed a community without depressive nodes. Our results suggested that among PTSD symptom nodes, “Flashback” and “Avoidance” were the two traumatic-event related but less associated with depressive symptoms, while “High alertness,” “Numb feeling,” and “Compunction and blame” were highly related to the depression. The results of community detection could act as a theoretical reference for the clinical diagnosis. Concerning the similarity of clinical symptoms between PTSD and depression (1), if firefighters were only observed with PTSD symptoms mentioned above which were closely related to depressive symptoms, the possibility of single depression diagnosis, instead of PTSD, should not be overlooked.

Additionally, “Mood circadian rhythm” and “Life emptiness” were the depressive nodes with the highest bridge EI in the respective community. As “Life emptiness” was also the node with the highest original EI, the data-driven results again proved its significance in the PTSD and depressive symptom network. Whereas, “Mood circadian rhythm” had a relatively low original EI but the highest bridge EI. Combined with the location of “Mood circadian rhythm” node in the total network (mainly connecting Community 2 and Community 3), we considered that this situation reflected the circadian rhythm of a series of depressive symptoms in Community 3. Given that the node with high bridge EI play a critical role in inducing the external community symptoms, and that Chinese firefighters are a high-risk, high-stress occupation with a lack of work routines, individualized psychological interventions based on the circadian rhythms of depressive symptoms are essential. Meanwhile, ensuring their healthy life routines can promote the recovery of depressive symptoms.

## 5. Limitations

Notwithstanding our study firstly demonstrated the PTSD and depression symptom network structure of Chinese firefighters, there are still some limitations need to be clarified. First, the current study was cross-sectional, thus the dynamic alterations and causality among symptom nodes could not be investigated. Second, firefighters recruited in our study were all male, which might limit the promotion of our results among female firefighters. As Cao et al. (67) demonstrated that the sex difference in symptoms connectivity of the PTSD symptom network among adolescents, we speculated that our results need to be further verified in female firefighters. Third, the symptom networks were generated specific to self-reported assessments applied in our study; therefore, the network structure was possibly affected by recall bias and participants’ social desirability. Additionally, self-reported assessments were applied rather than clinical interview; thus, atypical clinical features of PTSD and/or depression might be ignored.

## 6. Conclusion

To the best of our knowledge, the current study firstly demonstrated the network structure of PTSD and depressive symptoms among Chinese firefighters, identifying the central symptoms (i.e., Life emptiness, Fatigue, and Hopeless) and bridge symptoms (i.e., Numb feeling, Sad mood, and Compunction and blame). Meanwhile, the association between two clusters of symptoms was further clarified by the community detection analysis, indicating that core PTSD symptoms were “Flashback” and “Avoidance,” whereas “High alertness,” “Numb feeling,” and “Compunction and blame” were more relevant to depressive symptoms. Our work could act as an impetus for future studies investigating symptom networks of firefighters or other populations with high occupational risk (e.g., policemen, soldiers, etc.) as a method to verify the core symptoms that generalize across various populations with different culture backgrounds and those which are specific for certain populations. Moreover, central and bridge symptoms demonstrated by the current study can be possible targets for clinical intervention to treat firefighters who are suffering from PTSD and depressive symptoms.

## Data availability statement

The raw data supporting the conclusions of this article will be made available by the authors, without undue reservation.

## Ethics statement

The studies involving human participants were reviewed and approved by the Ethic Committee of the Second Xiangya Hospital of Central South University. The patients/participants provided their written informed consent to participate in this study.

## Author contributions

PC and LW: literature review and manuscript drafting. YZ, WM, and GZ: data acquisition. LZ and WL: study design and manuscript revision. All authors contributed to the article and approved the submitted version.

## Funding

This research was supported by Natural Science Foundation of China (No. 82171518 to LZ), the Natural Science Foundation of Hunan Province, China (No. 2020JJ5844 to LZ), and Technological Innovation Foundation of Central South University (No. 502801002 to WL).

## Conflict of interest

The authors declare that the research was conducted in the absence of any commercial or financial relationships that could be construed as a potential conflict of interest.

## Publisher’s note

All claims expressed in this article are solely those of the authors and do not necessarily represent those of their affiliated organizations, or those of the publisher, the editors and the reviewers. Any product that may be evaluated in this article, or claim that may be made by its manufacturer, is not guaranteed or endorsed by the publisher.
