# Supplementary material for: Post-traumatic stress disorder and depressive symptoms among firefighters: a network analysis
Source: Front Public Health. 2023 May 4;11:1096771. doi: 10.3389/fpubh.2023.1096771 (PMC10193951; doi:10.3389/fpubh.2023.1096771)
Supplement: Supplementary file 1 [file Data_Sheet_1.DOCX]

**Supplementary materials**

**Supplementary Table 1** Correlation matrix of the PC-PTSD-5 and SDS items

**Supplementary Figure 1** Bootstrapped confidence intervals of edge weights

**Supplementary Figure 2** Estimation of edge weight difference by bootstrapped difference test

**Supplementary Figure 3** Network structure of only-child firefighter sub-network

**Supplementary Figure 4** Network structure of non-only child firefighter sub-network

**Supplementary Table 2** Original centrality for sub-networks of only-child firefighters and non-only child firefighters

|  | PCPTSD1 | PCPTSD2 | PCPTSD3 | PCPTSD4 | PCPTSD5 | SDS1 | SDS2 | SDS3 | SDS4 | SDS5 | SDS6 | SDS7 | SDS8 | SDS9 | SDS10 | SDS11 | SDS12 | SDS13 | SDS14 | SDS15 | SDS16 | SDS17 | SDS18 | SDS19 | SDS20 |
| --- | --- | --- | --- | --- | --- | --- | --- | --- | --- | --- | --- | --- | --- | --- | --- | --- | --- | --- | --- | --- | --- | --- | --- | --- | --- |
| PCPTSD1 | 0.00 | 0.46 | 0.08 | 0.06 | 0.05 | 0.00 | 0.00 | 0.00 | 0.02 | 0.00 | 0.00 | 0.00 | 0.02 | 0.02 | 0.03 | 0.00 | 0.00 | 0.03 | 0.00 | 0.00 | 0.00 | 0.00 | 0.00 | 0.00 | 0.00 |
| PCPTSD2 | 0.46 | 0.00 | 0.11 | 0.00 | 0.05 | 0.00 | 0.00 | 0.03 | 0.00 | 0.00 | 0.00 | 0.00 | 0.00 | 0.00 | 0.00 | 0.00 | 0.00 | 0.04 | 0.00 | 0.00 | 0.00 | 0.00 | 0.00 | 0.01 | 0.00 |
| PCPTSD3 | 0.08 | 0.11 | 0.00 | 0.12 | 0.15 | 0.04 | 0.00 | 0.01 | 0.07 | 0.00 | 0.00 | 0.00 | 0.00 | 0.00 | 0.00 | 0.00 | 0.00 | 0.03 | 0.00 | 0.05 | 0.00 | 0.00 | 0.03 | 0.00 | 0.00 |
| PCPTSD4 | 0.06 | 0.00 | 0.12 | 0.00 | 0.27 | 0.14 | 0.00 | 0.06 | 0.01 | 0.00 | 0.00 | 0.00 | 0.06 | 0.00 | 0.05 | 0.00 | 0.00 | 0.00 | 0.01 | 0.00 | -0.01 | 0.00 | 0.05 | 0.02 | 0.00 |
| PCPTSD5 | 0.05 | 0.05 | 0.15 | 0.27 | 0.00 | 0.00 | 0.00 | 0.07 | 0.00 | 0.00 | 0.00 | 0.00 | 0.01 | 0.01 | 0.00 | 0.00 | 0.00 | 0.00 | 0.02 | 0.00 | 0.00 | 0.00 | 0.00 | 0.05 | 0.00 |
| SDS1 | 0.00 | 0.00 | 0.04 | 0.14 | 0.00 | 0.00 | 0.00 | 0.13 | 0.15 | -0.02 | 0.00 | 0.00 | 0.00 | 0.01 | 0.21 | 0.00 | 0.00 | 0.10 | 0.00 | 0.16 | 0.00 | 0.00 | 0.03 | 0.01 | 0.00 |
| SDS2 | 0.00 | 0.00 | 0.00 | 0.00 | 0.00 | 0.00 | 0.00 | 0.00 | 0.00 | 0.10 | 0.08 | -0.04 | 0.00 | 0.00 | 0.00 | 0.05 | 0.06 | 0.00 | 0.09 | 0.00 | 0.03 | 0.02 | 0.09 | 0.00 | 0.04 |
| SDS3 | 0.00 | 0.03 | 0.01 | 0.06 | 0.07 | 0.13 | 0.00 | 0.00 | 0.02 | 0.00 | 0.00 | 0.00 | 0.05 | 0.11 | 0.01 | 0.00 | 0.00 | 0.11 | 0.00 | 0.04 | 0.00 | 0.00 | 0.03 | 0.11 | 0.00 |
| SDS4 | 0.02 | 0.00 | 0.07 | 0.01 | 0.00 | 0.15 | 0.00 | 0.02 | 0.00 | 0.00 | 0.00 | 0.02 | 0.11 | 0.00 | 0.14 | 0.00 | 0.00 | 0.03 | 0.00 | 0.09 | 0.00 | 0.00 | 0.00 | 0.00 | 0.00 |
| SDS5 | 0.00 | 0.00 | 0.00 | 0.00 | 0.00 | -0.02 | 0.10 | 0.00 | 0.00 | 0.00 | 0.19 | 0.00 | 0.00 | 0.00 | 0.00 | 0.18 | 0.16 | 0.00 | 0.04 | 0.00 | 0.10 | 0.00 | 0.03 | 0.00 | 0.08 |
| SDS6 | 0.00 | 0.00 | 0.00 | 0.00 | 0.00 | 0.00 | 0.08 | 0.00 | 0.00 | 0.19 | 0.00 | 0.00 | 0.00 | 0.00 | 0.00 | 0.10 | 0.05 | 0.00 | 0.03 | -0.01 | 0.04 | 0.08 | 0.01 | 0.00 | 0.12 |
| SDS7 | 0.00 | 0.00 | 0.00 | 0.00 | 0.00 | 0.00 | -0.04 | 0.00 | 0.02 | 0.00 | 0.00 | 0.00 | 0.05 | 0.13 | 0.02 | -0.01 | 0.00 | 0.02 | -0.03 | 0.03 | 0.00 | -0.02 | 0.00 | 0.00 | 0.00 |
| SDS8 | 0.02 | 0.00 | 0.00 | 0.06 | 0.01 | 0.00 | 0.00 | 0.05 | 0.11 | 0.00 | 0.00 | 0.05 | 0.00 | 0.11 | 0.12 | 0.00 | 0.00 | 0.00 | 0.00 | 0.03 | 0.00 | 0.00 | 0.00 | 0.03 | 0.00 |
| SDS9 | 0.02 | 0.00 | 0.00 | 0.00 | 0.01 | 0.01 | 0.00 | 0.11 | 0.00 | 0.00 | 0.00 | 0.13 | 0.11 | 0.00 | 0.17 | 0.00 | 0.00 | 0.17 | 0.00 | 0.07 | 0.00 | 0.00 | 0.00 | 0.04 | 0.00 |
| SDS10 | 0.03 | 0.00 | 0.00 | 0.05 | 0.00 | 0.21 | 0.00 | 0.01 | 0.14 | 0.00 | 0.00 | 0.02 | 0.12 | 0.17 | 0.00 | 0.00 | 0.00 | 0.17 | 0.00 | 0.11 | 0.00 | 0.00 | 0.01 | 0.06 | 0.00 |
| SDS11 | 0.00 | 0.00 | 0.00 | 0.00 | 0.00 | 0.00 | 0.05 | 0.00 | 0.00 | 0.18 | 0.10 | -0.01 | 0.00 | 0.00 | 0.00 | 0.00 | 0.30 | 0.00 | 0.15 | 0.00 | 0.00 | 0.05 | 0.02 | 0.00 | 0.14 |
| SDS12 | 0.00 | 0.00 | 0.00 | 0.00 | 0.00 | 0.00 | 0.06 | 0.00 | 0.00 | 0.16 | 0.05 | 0.00 | 0.00 | 0.00 | 0.00 | 0.30 | 0.00 | -0.01 | 0.09 | 0.00 | 0.17 | 0.07 | 0.00 | 0.00 | 0.08 |
| SDS13 | 0.03 | 0.04 | 0.03 | 0.00 | 0.00 | 0.10 | 0.00 | 0.11 | 0.03 | 0.00 | 0.00 | 0.02 | 0.00 | 0.17 | 0.17 | 0.00 | -0.01 | 0.00 | 0.00 | 0.17 | 0.00 | 0.00 | 0.00 | 0.08 | 0.00 |
| SDS14 | 0.00 | 0.00 | 0.00 | 0.01 | 0.02 | 0.00 | 0.09 | 0.00 | 0.00 | 0.04 | 0.03 | -0.03 | 0.00 | 0.00 | 0.00 | 0.15 | 0.09 | 0.00 | 0.00 | 0.00 | 0.08 | 0.17 | 0.33 | 0.00 | 0.06 |
| SDS15 | 0.00 | 0.00 | 0.05 | 0.00 | 0.00 | 0.16 | 0.00 | 0.04 | 0.09 | 0.00 | -0.01 | 0.03 | 0.03 | 0.07 | 0.11 | 0.00 | 0.00 | 0.17 | 0.00 | 0.00 | 0.00 | 0.00 | 0.00 | 0.04 | 0.00 |
| SDS16 | 0.00 | 0.00 | 0.00 | -0.01 | 0.00 | 0.00 | 0.03 | 0.00 | 0.00 | 0.10 | 0.04 | 0.00 | 0.00 | 0.00 | 0.00 | 0.00 | 0.17 | 0.00 | 0.08 | 0.00 | 0.00 | 0.14 | 0.01 | 0.00 | 0.12 |
| SDS17 | 0.00 | 0.00 | 0.00 | 0.00 | 0.00 | 0.00 | 0.02 | 0.00 | 0.00 | 0.00 | 0.08 | -0.02 | 0.00 | 0.00 | 0.00 | 0.05 | 0.07 | 0.00 | 0.17 | 0.00 | 0.14 | 0.00 | 0.30 | 0.00 | 0.17 |
| SDS18 | 0.00 | 0.00 | 0.03 | 0.05 | 0.00 | 0.03 | 0.09 | 0.03 | 0.00 | 0.03 | 0.01 | 0.00 | 0.00 | 0.00 | 0.01 | 0.02 | 0.00 | 0.00 | 0.33 | 0.00 | 0.01 | 0.30 | 0.00 | 0.00 | 0.24 |
| SDS19 | 0.00 | 0.01 | 0.00 | 0.02 | 0.05 | 0.01 | 0.00 | 0.11 | 0.00 | 0.00 | 0.00 | 0.00 | 0.03 | 0.04 | 0.06 | 0.00 | 0.00 | 0.08 | 0.00 | 0.04 | 0.00 | 0.00 | 0.00 | 0.00 | 0.00 |
| SDS20 | 0.00 | 0.00 | 0.00 | 0.00 | 0.00 | 0.00 | 0.04 | 0.00 | 0.00 | 0.08 | 0.12 | 0.00 | 0.00 | 0.00 | 0.00 | 0.14 | 0.08 | 0.00 | 0.06 | 0.00 | 0.12 | 0.17 | 0.24 | 0.00 | 0.00 |

**Supplementary Table 1** Correlation matrix of the PC-PTSD-5 and SDS items

**
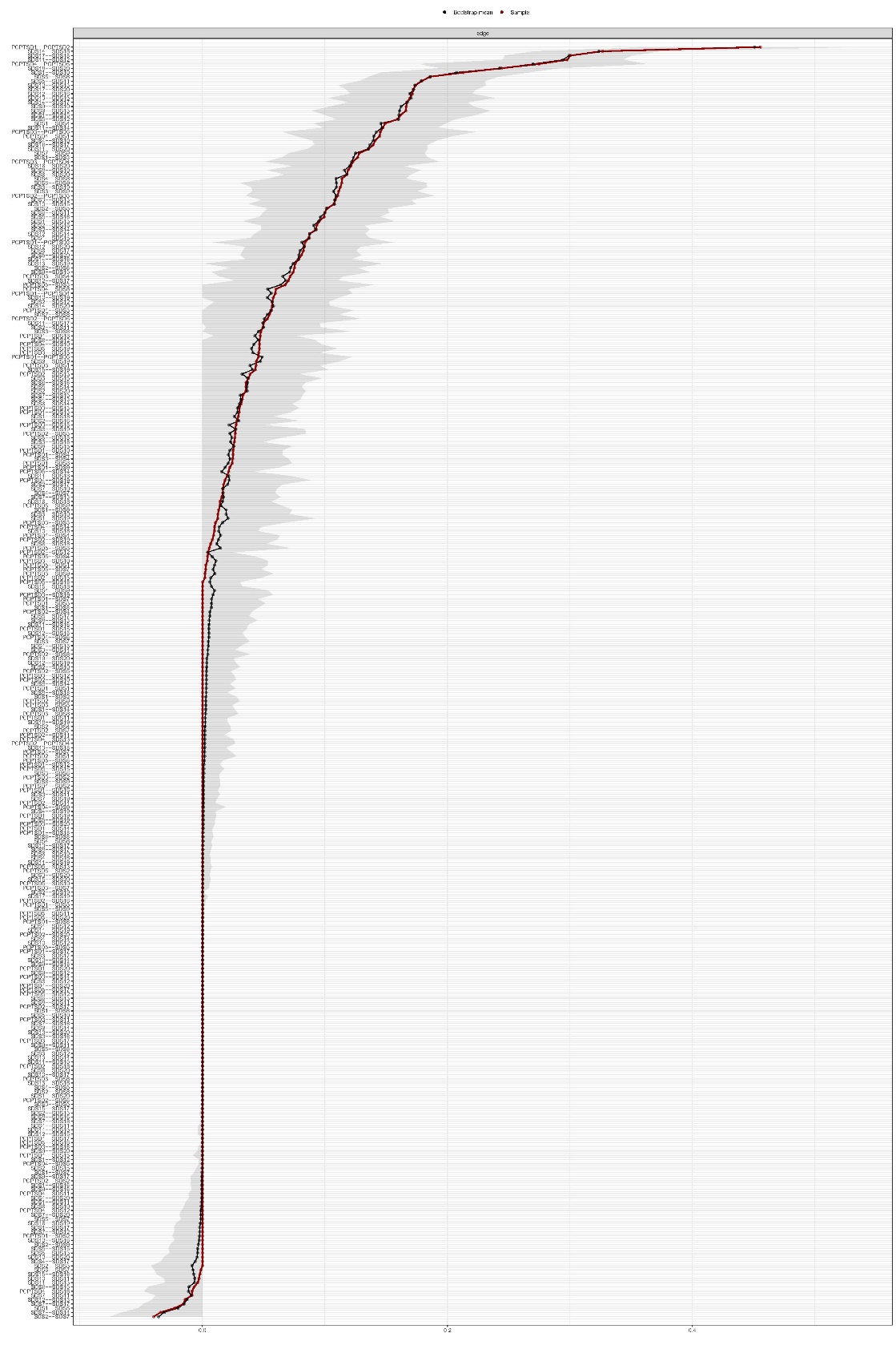
**

**Supplementary Figure 1.** Bootstrapped confidence intervals of edge weights

The black dots indicate the values of each edge weight, ordered from the highest to the lowest value. The gray area represents the 95% Confidence Intervals of edge weights, estimated with the non-parametric bootstrap procedure (by R *bootnet* package). Wide intervals suggest lower stability and narrow intervals mean higher stability.


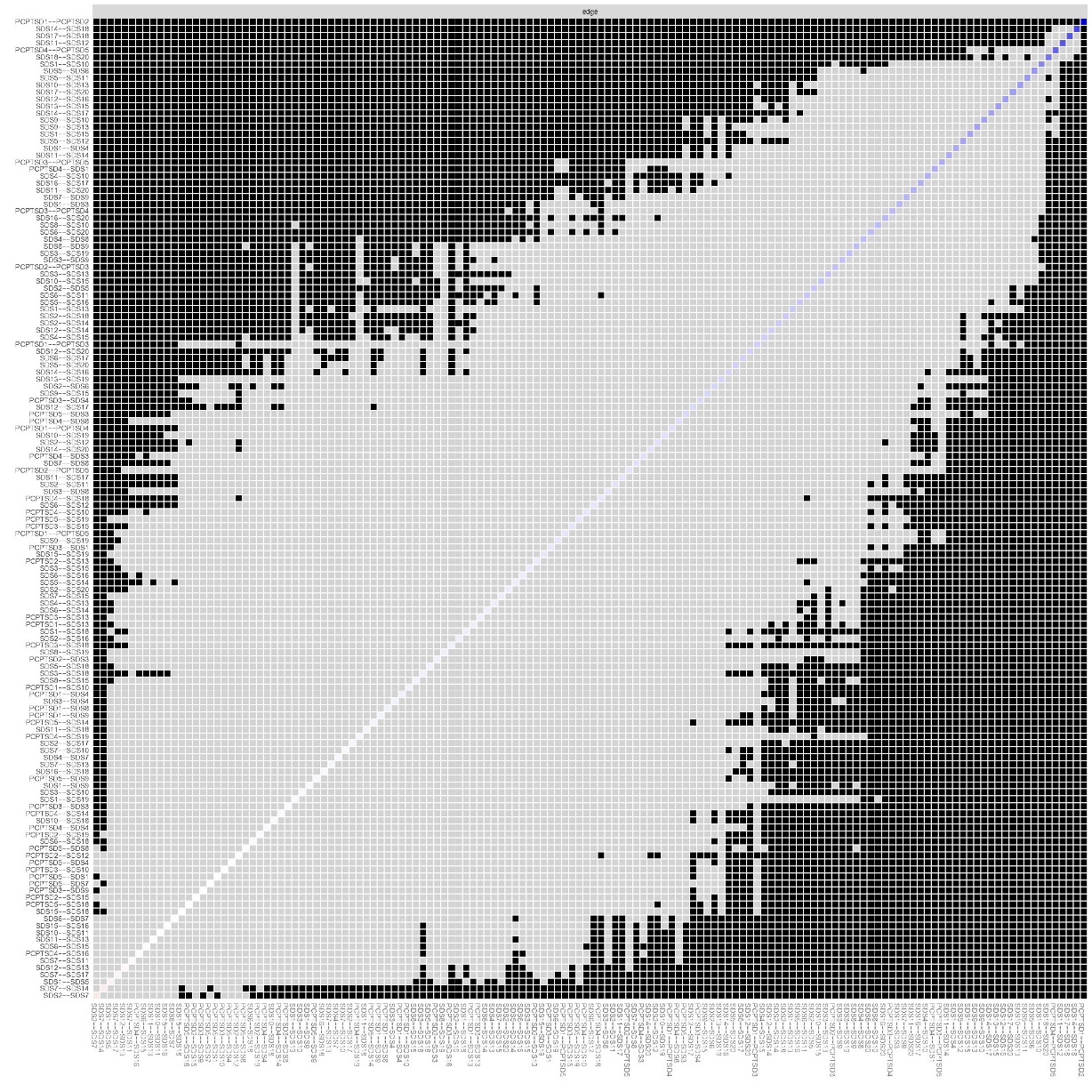


**Supplementary Figure 2.** Estimation of edge weight difference by bootstrapped difference test

Bootstrapped difference tests between edge weights in the network. Gray boxes suggest edges that do not differ significantly from one-another. Black boxes indicate edges with significant difference from one another (α = 0.05). Blue boxes in the edge-weight diagram indicate positive correlations.


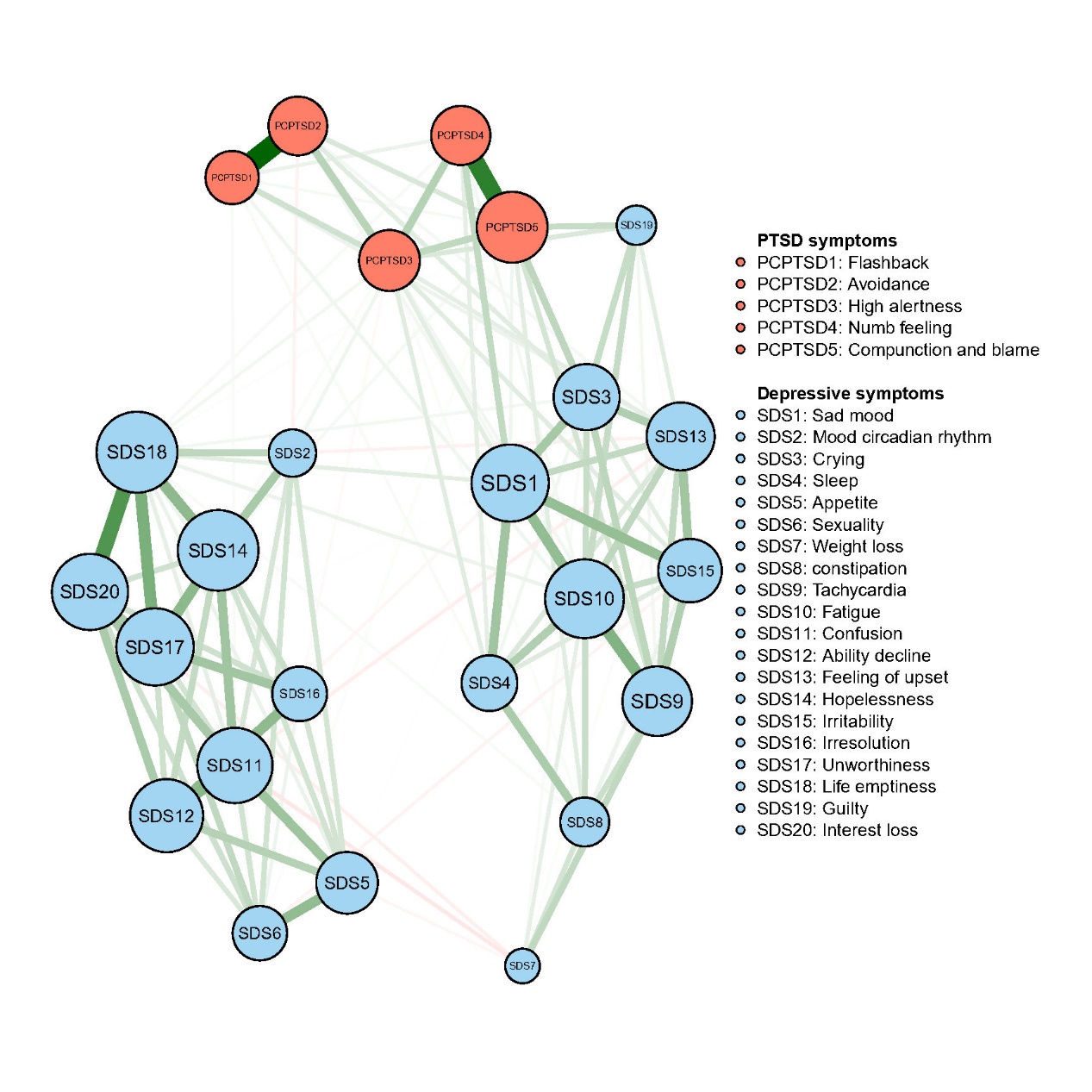


**Supplementary Figure 3** Network structure of only-child firefighter sub-network


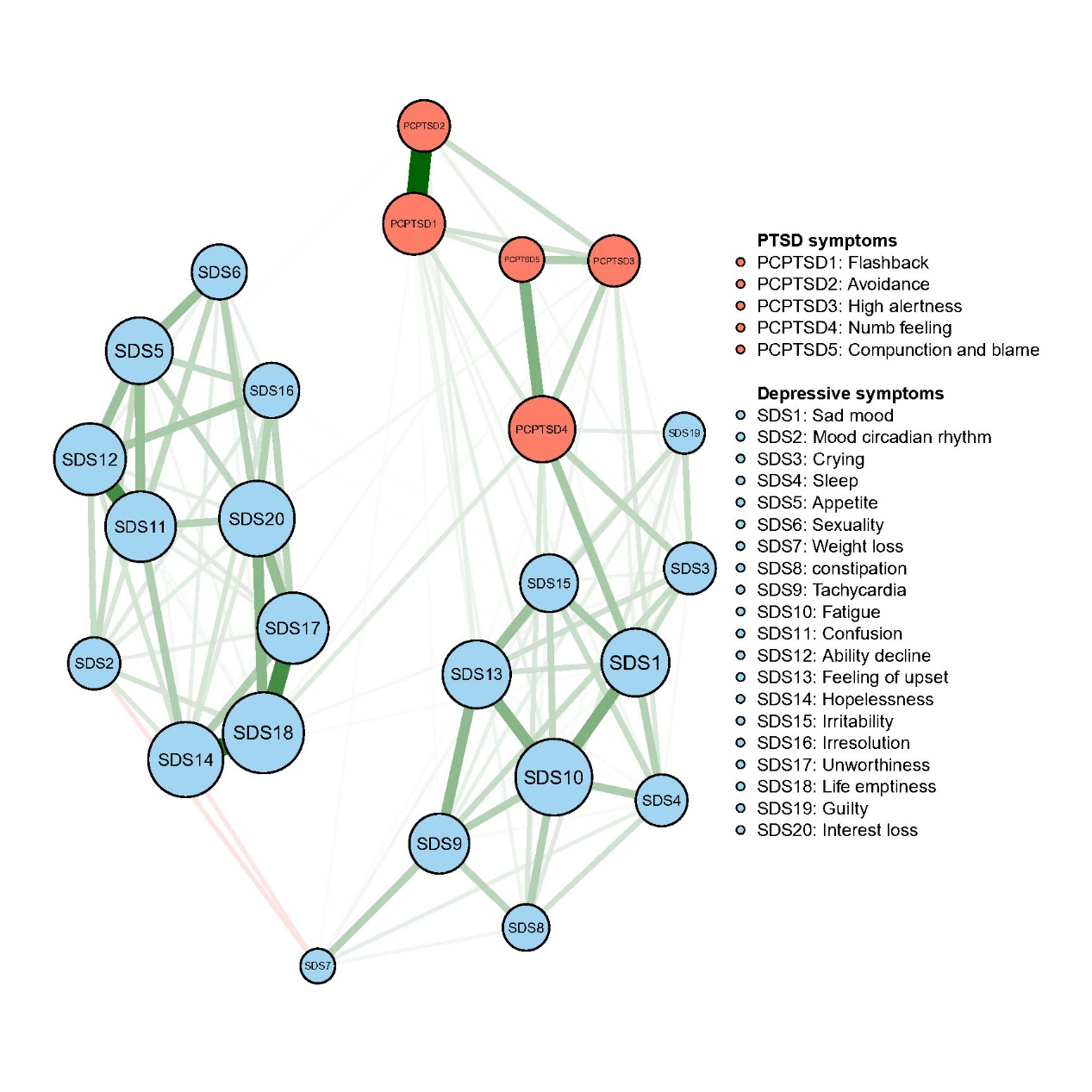


**Supplementary Figure 4** Network structure of non-only child firefighter sub-network

| **Symptom node** | **Abbr.** | **Original EI** | | **Test statistic C** | **P** |
| --- | --- | --- | --- | --- | --- |
|  |  | **Only-child firefighters** | **Non-only-child firefighters** |  |  |
| Flashback | PCPTSD1 | -0.595 | 0.187 | -0.133 | 0.85 |
| Avoidance | PCPTSD2 | -0.397 | -0.583 | 0.094 | 0.85 |
| High alertness | PCPTSD3 | -0.115 | -0.607 | 0.164 | 0.85 |
| Numb feeling | PCPTSD4 | -0.167 | 0.515 | -0.113 | 0.85 |
| Compunction and blame | PCPTSD5 | 0.680 | -1.122 | 0.468 | 0.20 |
| Sad mood | SDS1 | 0.952 | 0.641 | 0.112 | 0.85 |
| Mood circadian rhythm | SDS2 | -1.439 | -0.953 | -0.056 | 0.85 |
| Crying | SDS3 | 0.342 | -0.578 | 0.262 | 0.85 |
| Sleep | SDS4 | -0.410 | -0.575 | 0.090 | 0.85 |
| Appetite | SDS5 | -0.139 | 0.547 | -0.114 | 0.85 |
| Sexuality | SDS6 | -0.682 | -0.331 | -0.030 | 0.85 |
| Weight loss | SDS7 | -2.670 | -2.644 | 0.065 | 0.85 |
| constipation | SDS8 | -0.900 | -0.977 | 0.073 | 0.85 |
| Tachycardia | SDS9 | 0.591 | 0.048 | 0.170 | 0.85 |
| Fatigue | SDS10 | 1.259 | 1.311 | 0.023 | 0.99 |
| Confusion | SDS11 | 0.642 | 0.842 | -0.006 | 0.99 |
| Ability decline | SDS12 | 0.454 | 0.928 | -0.069 | 0.85 |
| Feeling of upset | SDS13 | 0.004 | 0.653 | -0.107 | 0.85 |
| Hopelessness | SDS14 | 1.394 | 0.814 | 0.172 | 0.42 |
| Irritability | SDS15 | -0.020 | -0.150 | 0.078 | 0.85 |
| Irresolution | SDS16 | -0.562 | -0.340 | -0.001 | 0.99 |
| Unworthiness | SDS17 | 0.895 | 0.900 | 0.038 | 0.85 |
| Life emptiness | SDS18 | 1.412 | 1.635 | -0.018 | 0.99 |
| Guilty | SDS19 | -1.598 | -1.381 | 0.010 | 0.99 |
| Interest loss | SDS20 | 1.067 | 1.221 | 0.001 | 0.99 |

**Supplementary Table 2** Original centrality for sub-networks of only-child firefighters and non-only child firefighters

**Note**: PCPTSD: The primary care PTSD screen for DSM-5; SDS: the self-rating depression scale; Abbr.: abbreviation; EI: expected influence
